# The Impact of Time-to-Intervention on Mortality and Functional Outcome in Acute Subdural Hematoma: A Systematic Review and Meta-Analysis

**DOI:** 10.3390/medsci14030412

**Published:** 2026-07-21

**Authors:** Bogdan Jabłoński, Wojciech Górecki, Justyna Fercho, Jacek Szypenbejl, Maksymilian Niemczyk, Oskar Chasles, Zuzanna Krasula, Zuzanna Wites, Mariusz Siemiński

**Affiliations:** 1 Scientific Circle of Neurotraumatology, Department of Emergency Medicine, Medical University of Gdańsk, 80-210 Gdańsk, Poland; bogdan.jablonski@gumed.edu.pl (B.J.);; 2 Department of Emergency Medicine, Medical University of Gdańsk, 80-210 Gdańsk, Poland; 3 Neurosurgery Department, 10th Military Research Hospital and PolyClinic SPZOZ in Bydgoszcz, 85-681 Bydgoszcz, Poland

**Keywords:** neurotraumatology, subdural hematoma, emergency medicine, timing, time to surgery, surgical treatment, TBI, traumatic brain injury, mortality, functional outcome

## Abstract

**Introduction:** Acute subdural hematoma (aSDH) represents one of the most lethal and debilitating forms of traumatic brain injury (TBI) encountered in neurosurgical emergency settings, carrying high mortality rates and severe long-term functional deficits. Historically, the timing of surgical decompression has been considered a critical modifiable prognostic factor. However, contemporary evidence regarding the relationship between time to surgery and clinical outcomes remains inconsistent. This systematic review and meta-analysis evaluates the impact of surgical timing on mortality and functional status in adult patients undergoing evacuation for acute subdural hematoma. **Methods:** A systematic review was conducted according to the PRISMA 2020 guidelines and registered in PROSPERO. A database search was performed across PubMed, Scopus, Embase and Web of Science for relevant studies published between 2009 and 2026. Peer-reviewed cohort studies and clinical trials including adults (>18 years) with CT- or MRI-confirmed aSDH were included. Data extraction focused on baseline characteristics, surgical timing thresholds, and primary outcomes of mortality and functional status assessed using the Glasgow Outcome Scale (GOS). Methodological quality was assessed using the Newcastle-Ottawa Scale. Relative risks (RR) and risk differences (RD) with 95% confidence intervals (CI) were calculated using a random-effects model. Results: The search identified 1815 records, of which six studies, including 926 patients, met the inclusion criteria. Meta-analysis found no statistically significant association between time to surgery and mortality (RR 1.01, 95% CI [0.69, 1.48], *p* = 0.96; RD −0.02, 95% CI [−0.19, 0.15]). High statistical heterogeneity was observed (I^2^ = 80%) across the included cohorts. Most studies indicated that patients who underwent rapid neurosurgical intervention frequently presented with significantly worse baseline neurological status, which acts as a primary confounding factor and drives poorer prognostic trends. **Conclusions:** While historical paradigms emphasize a 4h period for subdural hematoma evacuation, our meta-analysis indicates that a straightforward linear relationship between time to intervention and improved survival or functional outcome is absent in contemporary clinical studies. Surgical timing thresholds must be interpreted cautiously, as the decision-making process is heavily guided by clinical triage bias, where rapidly deteriorating patients are prioritized for urgent decompression. Crude timing thresholds do not reflect a lack of causal benefit of early intervention but rather highlight the profound confounding by indication embedded in contemporary neurotrauma literature. Future multicenter investigations adjusting for admission severity, pupillary reactivity, associated intracranial pathology and other confounding factors are crucial to isolate the true prognostic value of surgical timing in aSDH.

## 1. Introduction

Traumatic acute subdural hematoma (aSDH) is one of the most severe forms of traumatic brain injury (TBI). Despite major advancements in emergency medicine and neurosurgery, diagnostic imaging, and pre-hospital emergency medical systems, the overall mortality rate remains high, typically ranging from approximately 10% to over 70%, with many survivors suffering long-term functional deficits [1].

Historically, the time from injury to surgical intervention has been considered a critical modifiable factor influencing patient outcomes. Since the landmark study by Seelig et al. in 1981, which reported a dramatic mortality reduction (from 90% to 30%) for comatose patients treated within four hours of injury [2], the “four-hour rule” has remained a cornerstone of trauma management and a benchmark for trauma system performance. The biological rationale is the prompt relief of intracranial mass effect to prevent secondary ischemic injury and irreversible brainstem compression [3,4].

However, contemporary literature presents inconsistent evidence regarding the role of surgical timing. While some research indicates that a better functional outcome is associated with earlier intervention [5]; other studies have failed to identify a consistent association between timing and survival. Paradoxically, some retrospective cohorts have reported that patients operated on more quickly often experience higher mortality rates, which can probably be attributed to bias in patient qualification for surgery, i.e., patients with rapidly deteriorating neurological status are often prioritized for emergency operation [6,7,8,9].

Given these discrepancies across decades of research and the lack of consensus across patient subgroups for various age groups and clinical presentations, a comprehensive synthesis is necessary. This systematic review and meta-analysis aim to evaluate the independent impact of time-to-intervention and surgical technique on mortality and functional recovery while accounting for clinical and radiological confounders.

## 2. Methods

### 2.1. Search Strategy and Protocol

This systematic review was conducted in accordance with the Preferred Reporting Items for Systematic Reviews and Meta-Analyses (PRISMA) 2020 guidelines [10]. The study protocol was registered on PROSPERO (CRD420261381945).

A comprehensive literature search was performed across four electronic databases: PubMed, Scopus, Web of Science, and Embase. The studies published between January 2009 and January 2026 were included in the screening process. The search strategy was restricted to peer-reviewed articles published in the English language. Non-English publications were excluded due to resource limitations regarding institutional medical translation services. Furthermore, gray literature databases, preprint servers and clinical trial registries were not searched. This boundary was maintained to ensure that all pooled clinical and radiological data had undergone formal, rigorous peer review, which is crucial given the high potential for methodological variation and baseline confounding in retrospective neurotrauma literature. The search was performed using the phrase (“epidural hematoma” OR “subdural hematoma” OR “EDH” OR “SDH”) AND (“Time-to-Treatment” OR “time to surgery” OR “timing” OR “delay”) AND (“outcome” OR “mortality” OR “neurological”).

The retrieved papers were uploaded to Synthos.app, a program that combines search results downloaded from multiple scientific databases and, by using the DOI identifier as a key value, finds papers that exist in more than one database. If a paper has more than one instance in the combined dataset, each entry except the first one is counted as a duplicate. All entries with the same key value (DOI) are also compared to each other to unify the paper’s metadata (title, abstract, authors, keywords, year, journal, PMID, direct URL). The initial screening of titles and abstracts, followed by the full-text assessment, was conducted independently by two reviewers utilizing Synthos.app (W.G. and M.N.). Disagreements at any stage of the process were resolved by a third author (J.F.), who made the final decision following a detailed discussion with the two primary reviewers.

### 2.2. Inclusion and Exclusion Criteria

The studies were included if they involved adult patients (>18 years) with traumatic acute subdural hematoma (aSDH) confirmed by CT or MRI. Eligible studies reported the patients’ outcomes in the form of mortality or functional outcome (GOS, mRS). The study designs included randomized controlled trials, prospective or retrospective cohort studies, case-control studies, multicenter studies, and other observational studies, peer-reviewed, published in English, that provided extractable data for all treatment groups.

The studies were excluded if they involved pediatric populations (<18 years), non-traumatic, chronic, spontaneous, or subacute subdural hematomas, or mixed intracranial hemorrhages. Studies that did not compare groups based on time-to-treatment, those without clinically relevant outcomes, or with insufficient data for extraction were excluded. Case reports or series with fewer than 10 patients, reviews, editorials, letters, comments, animal studies, and non-English publications were also excluded.

### 2.3. Data Extraction

From each included study (and their respective Supplementary Materials where such were available), data were extracted independently by two authors (Z.K. and M.N.). Discrepancies were resolved by a third reviewer (B.J.). The extracted data included: study identification details (DOI, link, first author and year of publication), country of origin, and study design (e.g., randomized controlled trial, retrospective study, or cohort study). Baseline demographic and clinical characteristics were extracted, including total number of patients, mean age, sex distribution, mean Glasgow Coma Scale (GCS) score, presence of pupillary abnormalities, mean hematoma thickness, midline shift (MLS), and hematoma volume, with corresponding standard deviations and sample sizes where available. Outcome measures included mortality in all treatment groups, functional outcomes assessed using the Glasgow Outcome Scale (GOS), and reported complications with their respective frequencies. All of the aforementioned variables were extracted separately for each treatment group, provided that the respective data were available in the paper or Supplementary Materials.

### 2.4. Risk of Bias

The risk of bias in the included observational studies was independently assessed by two reviewers using the Newcastle–Ottawa Scale (NOS). This tool evaluates studies across three domains: selection of study groups, comparability of groups, and ascertainment of outcomes. Each study received a score ranging from 0 to 9, with higher scores indicating a lower risk of bias. Discrepancies between reviewers were resolved through discussion or consultation with a third reviewer. Studies with NOS scores of ≥7 were considered at low risk of bias, scores of 4–6 indicated moderate risk, and scores < 4 indicated high risk.

Although a funnel plot analysis was conducted and demonstrated visual asymmetry, this finding should not be interpreted as definitive evidence of publication bias. Tests for funnel plot asymmetry are underpowered and unreliable when a meta-analysis includes fewer than 10 studies. Because only six studies were included, the analysis lacked sufficient statistical power to distinguish true publication bias from random variation.

### 2.5. Statistical Analysis

Statistical analyses were performed using Review Manager version 5.4. To assess the impact of time-to-intervention on patient outcomes, two primary effect measures were utilized: Risk Ratio (RR) and Risk Difference (RD), both reported with 95% confidence intervals (CI). To account for zero cells or boundary proportions (such as the 100% poor-outcome event rate in the compressed late arm (*n* = 5) of Chen et al. [5]), a standard fixed continuity correction of 0.5 was automatically applied to all cells of the respective study’s 2 × 2 table. This correction prevents mathematical indeterminacy (division by zero) and allows the estimation of standard errors and relative risks. The RD was specifically calculated to provide a clinical estimate of the absolute change in the probability of mortality or unfavorable functional outcomes (2 or 3 points on the Glasgow Outcome Scale, excluding death, which was analyzed separately). Inter-study heterogeneity was evaluated using the I^2^ statistic. A random-effects model (Mantel-Haenszel method) was used as the primary analysis to account for anticipated clinical and methodological diversity across the included neurosurgical cohorts. To explore the influence of specific “time-to-intervention” thresholds, subgroup analyses were conducted. Sensitivity analyses were performed by systematically excluding individual studies to ensure the robustness of the pooled estimates and to identify potential sources of significant heterogeneity. Statistical significance was defined as a two-sided *p*-value < 0.05.

## 3. Results

### 3.1. Search Results

**Figure 1:**
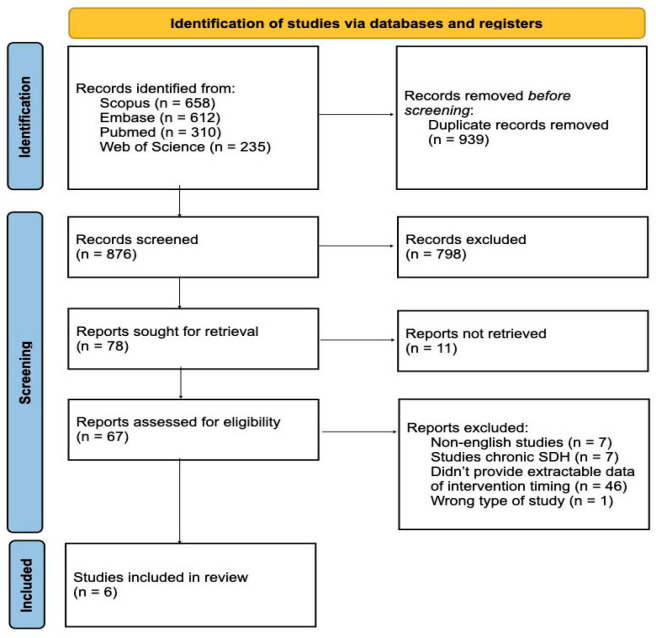
PRISMA flow diagram.

The initial search identified 1815 articles. After removing duplicates, 876 articles were subjected to title and abstract screening, where 798 were excluded, and out of the remaining 78 articles, 6 were possible to be retrieved and reported sufficient data to be included in this review [Table 1]. The search process is summarized in the PRISMA flow diagram [Figure 1].

### 3.2. Risk of Bias

The methodological quality of the included studies reached 7–8 points, which nominally indicates a moderate-to-low risk of bias [Table 2]. However, this overall summary score requires a highly cautious interpretation, as it frequently masks deep-seated structural challenges within the literature’s comparability domain. While the studies achieving full marks for comparability successfully implemented multivariate models, the cohorts that failed to adjust for confounders yielded crude estimates heavily distorted by clinical triage bias (selection bias). Conversely, the select studies that did successfully isolate and adjust for these confounding variables did so by enforcing highly restrictive clinical exclusion criteria, which inherently curtails their generalizability to the broader, heterogeneous aSDH population. Consequently, despite the high nominal quality scores, the true certainty of the pooled evidence is structurally constrained by this unavoidable trade-off between internal validity and external generalizability.

### 3.3. Mortality

**Figure 2:**
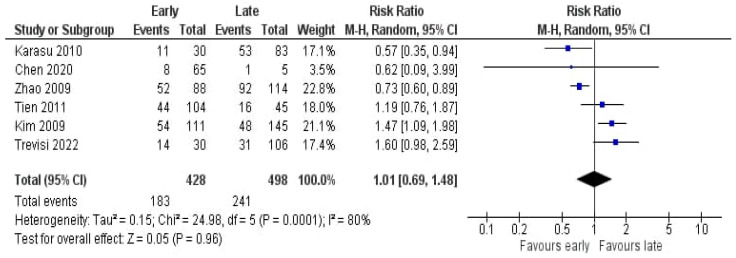
A forest plot evaluating the Risk Ratio (RR) for mortality [1,5,6,7,9,11].

**Figure 3:**
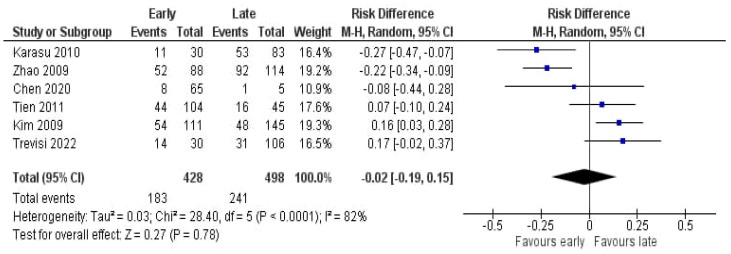
A forest plot evaluating the Risk Difference (RD) for mortality [1,5,6,7,9,11].

Early interventions were defined as those performed within 4h from the accident and late interventions as those performed after this threshold. A meta-analysis of six studies involving 926 total participants did not demonstrate a statistically significant difference in mortality between early and late interventions (RR 1.01, 95% CI [0.69, 1.48], *p* = 0.96; RD −0.02, 95% CI [−0.19, 0.15], *p* = 0.78). Significant heterogeneity was observed (I^2^ = 80%), suggesting that the timing of the intervention may be influenced by study-specific factors or clinical settings [Figure 2 and Figure 3].

### 3.4. Poor Outcomes

**Figure 4:**
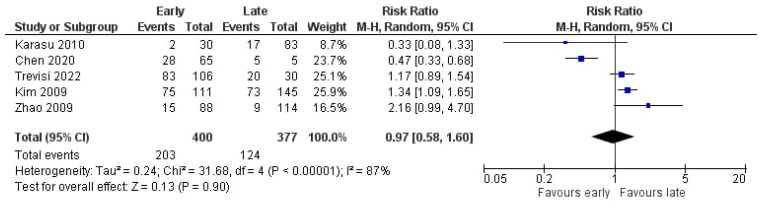
A forest plot evaluating the Risk Ratio (RR) for poor functional outcomes [1,5,7,9,11].

**Figure 5:**
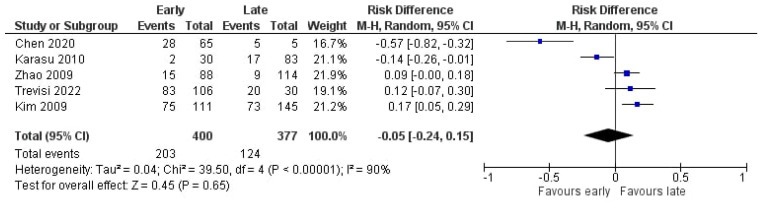
A forest plot evaluating the Risk Difference (RD) for poor functional outcomes [1,5,7,9,11].

While the pooled analysis showed no significant difference in poor outcomes (RR 0.97, *p* = 0.90), the results were characterized by extreme heterogeneity (I^2^ = 87%). Individual studies showed conflicting results, suggesting that the effect of timing is highly dependent on other clinical variables not captured in the primary analysis [Figure 4 and Figure 5].

## 4. Discussion

Acute subdural hematoma is the most common intracranial mass lesion and one of the most severe consequences of traumatic brain injury (TBI), associated with high mortality rates and neurological deficits. Although timely neurosurgical assessment and intervention are widely considered essential, the optimal timing of the operation remains controversial.

In this systematic review and meta-analysis, we found no statistically significant difference in mortality or poor functional outcome between early and late surgical intervention. However, substantial heterogeneity across included studies suggests that the relationship between the time from injury to surgical intervention and outcomes is not straightforward and is influenced by various factors [2,5,6,7,9,11] as shown in Table 3. It is important to note that this absence of statistical significance should not be interpreted as evidence that timing is clinically irrelevant.

A landmark study conducted by Seelig et al. in 1981 concluded that mortality could be reduced threefold if the patient is subjected to operational treatment within four hours of the accident, compared to patients who were operated on after this threshold (30% mortality in the early intervention group and 90% in the group operated on after four hours since the accident) [2]. This study became foundational in the matter of intervention timing in aSDH and the 4h threshold became a widely adopted standard in other studies on this topic as well as a clinical paradigm taught in textbooks and widely adopted in clinical practice [12].

On the other hand, more contemporary studies failed to reproduce the substantial reduction in mortality of Seelig et al. and are much more nuanced when it comes to drawing conclusions about optimal intervention timing, some saying that timing is not at all related to outcomes [13,14,15] and some showing a correlation that despite being statistically significant favors early intervention in some studies and late intervention in others [2,5,6,7,8,9]. Due to differences in the design of the included studies, we can analyze how the results differ depending on the studied population and impact on confounding variables. Kim et al. analyzed a relatively large group of patients with aSDH (*n* = 256) and did not apply any strict exclusion criteria, which improves the generalizability of the results. While looking for clinical variables that are significantly related to the outcomes, they found that time-to-surgery (TTS) was a predictor of mortality, but the percentage of deaths was bigger in the group operated on later than 4h after the trauma. They attribute this relation to a tendency to send severe cases of TBI quicker to the neurosurgery unit than the milder cases. Therefore, patients who had a less favorable clinical presentation and bigger chance of death because of other factors are more often found in the early intervention cohort. Thus, time-to-intervention functions predominantly as a marker of injury severity rather than an independent determinant of outcome; hence, the pooled estimates may obscure clinically meaningful differences [9].

Among the studies that demonstrated the effect of TTS on functional outcomes, Chen et al. [5] performed a multivariate analysis of 70 patients with a mean age of 50.9 (±14.6) and GCS of 5.9 (±1.1) in which TTS emerged as the only variable correlated with functional recovery in a statistically significant way. Specifically, the mean TTS was significantly shorter in the functional recovery group than in the poor-recovery group (145.5 ± 27.0 vs. 181.9 ± 54.5 min, *p* = 0.002). Using a Receiver Operating Characteristic (ROC) analysis to identify an optimal surgical timing threshold, the authors reported a cutoff of 177.5 min (2 h and 57.5 min), which demonstrated a sensitivity of 51.5% and a specificity of 91.9% for predicting functional recovery. Those results, although certainly insightful when considering a certain type of patient, should be interpreted with caution, as from the patients’ database were excluded those of an age greater than 70, with GCS outside the range of 4–8 (combined with bilateral pupil dilatation) and those with brain damage other than aSDH. This relatively homogenous group of subjects with overall quite severe clinical parameters proves that in cases of low GCS, time is crucial, but the strict exclusion criteria limit the generalizability of this study.

Several explanations have been proposed for those seemingly paradoxical results. The most common one is that patients with the worst prognosis are operated on faster and therefore increase the mortality rates in the early intervention group. Those who are in a good enough state to be able to wait longer consequently show lower chances of mortality and a higher likelihood of functional recovery [6,8]. Across the included studies, several factors were associated with mortality and functional outcome. The most common ones are: GCS, midline shift, occurrence of intraoperational acute brain swelling, age, pupil abnormalities (in size and/or reactivity, as a sign of transtentorial herniation), hematoma size measured on CT [Table 3].

Similar findings have been reported in other studies. Trevisi et al. analyzed a cohort of elderly patients (≥70 years) with aSDH and found that timing of the surgery did not significantly influence survival or functional outcomes, even after stratification for preoperative GCS. Early intervention was more frequently performed in patients with worse clinical and radiological presentation, suggesting a selection bias related to initial injury severity. Among the analyzed variables, preoperative midline shift was found to be the strongest predictor of outcome [7].

Another study by Karasu et al. analyzed patients with aSDH undergoing surgery within and after 4 h from trauma. In their cohort, patients operated within 4 h had significantly lower mortality (36.7% vs. 63.8%) and higher rates of functional recovery (56.7% vs. 15.7%). Based on these results, the authors underscored the need for urgent surgical intervention. However, the study did not clearly adjust for differences in initial clinical condition between the early and delayed surgery groups. Other prognostic factors such as GCS, pupil abnormalities, and age were also identified as important determinants of outcome [1].

Similar variability in findings was also observed among studies identified during the screening process but not included in the quantitative analysis due to insufficient data. Overall, these studies failed to demonstrate a clear effect of time-to-surgery on outcomes, further underscoring the complexity of this relationship.

### 4.1. Breaking Down the Timeline

Most studies simply count the amount of time from injury to surgical incision, but this variable can be broken into smaller ones to bring some nuance to the topic. Tien et al. distinguished prehospital time (PHT) by counting from the moment of injury to the arrival at the emergency department, trauma room to surgery (TRS) time from the arrival in the trauma room to surgical incision, and surgical time as the sum of the two aforementioned times [6]. Statistical analysis showed that although the impact of TRS on mortality did not reach statistical significance, a lower PHT was related to lower mortality, reinforcing the notion of the “Golden Hour” as a paradigm according to which the arrival of the patient at the emergency department during the first hour after injury has a beneficial effect on their outcome. A surgical time below the threshold of four hours was once again correlated with higher mortality, but the authors attribute this to the more severe presentations of the patients undergoing early surgery. However, in addition to medical emergencies, logistical factors such as operating room availability and neurosurgical team readiness may also lead to time-to-intervention vs. condition mismatch.

### 4.2. Type of Operation

Although the main topic of the analyzed studies was the impact of surgical timing on outcomes, they often also touched on the topic of types of surgery influencing mortality and functional results. The two most commonly performed surgical procedures in aSDH are craniotomy and decompressive craniectomy. The latter is universally associated with worse chances of recovery and higher mortality, attributed to the usage of this surgical method in patients who manifested an incidence of the aforementioned acute intraoperational brain swelling, which is a significant predictor of worse outcomes [9]. Although this explanation is widely accepted by researchers, not all studies are able to show statistical significance of the difference the operation type makes on the outcome [15]. A third, although less frequently mentioned method, is burr hole evacuation. Graham et al. analyzed the usage of this method in an elderly population of patients with a clinical presentation stable enough to afford waiting 48h from time of injury (GCS ≥ 13, maximal hematoma thickness of >10 mm and/or a midline shift size of >5 mm), which allowed further monitoring and stabilizing of the patient and showed significantly better outcomes than in the compared craniotomy group where surgery was performed within 24h from injury [8]. Although this is a very specific demographic that is not representative of the general population of patients requiring urgent evacuation from aSDH, it is worth noting that when the patient’s parameters allow delaying the operation, it may be beneficial to take advantage of this possibility, especially in such a vulnerable population as the elderly.

## 5. Limitations

This study has several limitations, which did not permit quantitative analysis of many variables, including but not limited to: GCS, rate of pupil non-reactivity and dilation, ICP and measurements of the hematoma on CT. The reason for those shortcomings is the scarce reporting of clinical data in the studies included in this meta-analysis. Even if certain parameters were reported at all, they were not usually broken up into timing categories, which made statistical analysis impossible and made us resort to the use of descriptive analysis of the most important findings of the studies. A further obstacle in our comparisons was the non-uniform categorization into timing categories across studies. Although most of them used the ’standard’ threshold of four hours, not all of them adopted this approach, leading to their exclusion from certain calculations as a measure of guaranteeing methodological accuracy. Some studies pointed out as their limitation the selectiveness of their cohorts, which limits the generalizability of their findings and causes the high heterogeneity of our meta-analysis, which signifies that the outcome of our calculations was heavily influenced by distinct factors specific to each facility conducting the corresponding study. All of them were single-center studies which further caused their results to be subjected to selection bias. Given those discrepancies across years of research, geographical settings of the research centers, studied populations and ways to present their patients’ clinical data, there is a clear need to conduct new studies in a standardized manner when it comes to reporting data. To allow future meta-analyses to quantitatively analyze variables related to the incidence and treatment of aSDH the researchers would need to either clearly state the said data both for the general population and for each analyzed subgroup, or attach the raw anonymized data of their patients as Supplementary Material, allowing the extraction and calculation of whatever data is needed. A major limitation of this study is our reliance on crude, unadjusted data extracted from the primary literature, as most contemporary studies failed to provide risk-adjusted effect sizes stratified by time-to-intervention. Because time behaves predominantly as an indicator of baseline injury severity rather than an independent variable, our pooled summary estimate must be interpreted as a reflection of current clinical triage realities rather than a true causal metric of surgical timing.

## 6. Conclusions

The available evidence does not clearly support time to surgery as an independent predictor of mortality or functional outcome in patients with aSDH. Outcomes seem to depend more on initial clinical and radiological conditions than on time to surgery alone, with factors such as preoperative GCS, midline shift, pupil abnormalities, and age being particularly important. It is necessary to clarify that these findings should not be interpreted as justification for intentionally delaying surgery in patients with established surgical indications or neurological deterioration.

Future studies should pay more attention to how time to surgery is defined, with a clearer distinction between pre-hospital time, diagnostic delays, and in-hospital time before surgery. It would also be helpful to analyze patients based on their initial clinical condition. Adjustment for factors such as GCS, midline shift, and pupil abnormalities may be essential to identify patient subgroups in which surgical timing has the greatest impact on outcomes and provide an understanding of the relationship between timing and outcomes in various clinical scenarios.

From a clinical perspective, these findings support an individualized approach, in which decisions about surgical treatment are made in the context of overall neurological status and imaging findings rather than a rigid time threshold alone.

## Figures and Tables

**Table 1:** Study characteristics comparison.

	Year	Patients (n)	Mean Age	Mean GCS	Mortality (%)	Functional Recovery (%)	How Was TTS ^1^ Defined	When Was the Outcome Measured	Conclusions
Chen et al. [5]	2020	70	49.4	5.7	12.9	52.9	the documented time of injury notification to the initiation of surgery	at least 1 year after the injury	TIS ^2^ is crucial for the functional recovery of TASDH ^3^ patients who underwent surgery. The threshold time for functional recovery of comatose and surgically treated TASDH patients was 2 h and 57.5 min in our study.
Karasu et al. [1]	2010	113	32	N/A	56.6	26.5	time interval between the trauma and operation	At 1-year follow up	In patients with PASH ^4^, the GCS scores, abnormal pupil reaction on admission, age, and timing of the operation are all prognostic determinants. A rise in intracranial pressure should be kept under control; CCT ^5^ has to be taken as soon as possible, and patients should be operated upon urgently if there is an indication for surgery.
Kim et al. [9]	2009	256	51.8	N/A	39.8	42.2	time elapsed from accident to surgery	3 months after admission or at death	Intraoperative ABS ^6^, preoperative pupillary abnormalities and GCS score were predictors for mortality of surgically treated traumatic aSDH. The age, mechanism of injury, intraoperative ABS, preoperative pupillary abnormalities and GCS score were independently significant predictors for functional recovery of surgically treated traumatic aSDH.
Tien et al. [6]	2011	149	44.7	7.1	40	N/A	number of minutes elapsed from injury to time that craniotomy commenced in the operating room	At discharge	Rapid transport of patients with traumatic subdural hematomas to the hospital is associated with decreased mortality […] We feel that this effect may be mediated both by reduced airway manipulation in the field and by a rapid assessment by the trauma and neurosurgical teams, to determine which patients require immediate craniotomy.
Trevisi et al. [7]	2022	136	78.5	10.3	33	24.3	from A&E arrival to the starting time of the surgical procedure	At discharge	In patients ≥ 70 years old, operated on for a post-traumatic aSDH, the main factors associated with the timing of surgery were GCS and radiological findings such as aSDH thickness and midline shift, with preoperative midline shift emerging as the only factor associated with survival and functional outcome at the multivariate analysis. The timing of surgery neither influenced the survival nor functional outcome. However, critical patients were almost always treated in an ultra-early timing.
Zhao et al. [11]	2009	202	47	4.8	71.3	19	time from injury to operative intervention	At 6-month follow up	Timely and aggressive evacuation of aSDH is critical once the indications for surgery come into existence, although not every patient with aSDH needs to be subject to operation.

^1^ TIS—time to surgery; ^2^ TIS—time from injury to surgery; ^3^ TASDH—traumatic acute subdural hematoma; ^4^ PASH—posttraumatic acute subdural hematoma; ^5^ CCT—computerized cranial tomography; ^6^ ABS—acute brain swelling. N/A—not available.

**Table 2:** Quality assessment of included studies using Newcastle–Ottawa Scale (NOS). Each star stands for one point in the corresponding domain.

Study (Author, Year)	Selection	Comparability	Outcome	Total Points	Quality
Zhao, 2009 [11]	⭐⭐⭐	⭐	⭐⭐⭐	7	Moderate-to-high
Chen, 2020 [5]	⭐⭐⭐	⭐⭐	⭐⭐⭐	8	High
Karasu, 2010 [1]	⭐⭐⭐	⭐	⭐⭐⭐	7	Moderate-to-high
Kim, 2009 [9]	⭐⭐⭐	⭐⭐	⭐⭐	7	Moderate-to-high
Tien, 2011 [6]	⭐⭐⭐	⭐⭐	⭐⭐	7	Moderate-to-high
Trevisi, 2022 [7]	⭐⭐⭐	⭐⭐	⭐⭐	7	Moderate-to-high

**Table 3:** Comparison of variables statistically analyzed for prognostic value of mortality and functional outcome.

Variable	Chen et al.	Karasu et al.	Kim et al.	Tien et al.	Trevisi et al.	Zhao et al.
Age	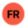	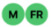	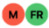	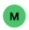	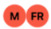	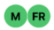
GCS	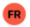	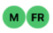	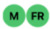	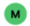	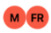	N/A
Intraoperative ABS	N/A	N/A	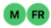	N/A	N/A	N/A
Pupillary abnormalities	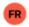	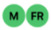	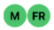	N/A	N/A	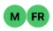
Eloquence of lesion	N/A	N/A	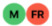	N/A	N/A	N/A
Midline shift	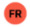	N/A	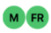	N/A	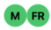	N/A
TTS	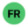	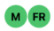	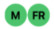	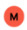	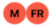	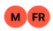
Mechanism of injury	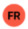	N/A	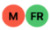	N/A	N/A	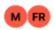
Volume of hematoma	N/A	N/A	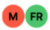	N/A	N/A	N/A
Thickness of hematoma	N/A	N/A	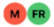	N/A	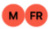	N/A
Sex	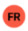	N/A	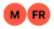	N/A	N/A	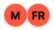
Mannitol dosage	N/A	N/A	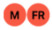	N/A	N/A	N/A
Prehospital time	N/A	N/A	N/A	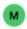	N/A	N/A
Trauma room to surgery time	N/A	N/A	N/A	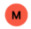	N/A	N/A
Injury severity score	N/A	N/A	N/A	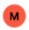	N/A	N/A
CT findings of subarachnoid hemorrhage	N/A	N/A	N/A	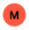	N/A	N/A
CT findings of herniation	N/A	N/A	N/A	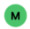	N/A	N/A
Postop ICP	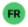	N/A	N/A	N/A	N/A	N/A

M—related to mortality; FR—related to functional recovery; N/A—relation to outcome not assessed in the given study; 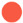
—not related to outcome or related in a statistically **non**significant way; 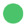
—related to outcome in a statistically significant way.

## Data Availability

The original contributions presented in the study are included in the article/Supplementary Material. Further inquiries can be directed to the corresponding author.

## Supplementary Materials

The following supporting information can be downloaded at: https://www.mdpi.com/article/10.3390/medsci14030412/s1. PRISMA manuscript checklist.
